# Carbon monoxide poisoning: a clinical case report

**DOI:** 10.1186/s12245-024-00777-0

**Published:** 2024-12-18

**Authors:** Deimante Baksevice, Aida Mankute-Use, Austeja Bernotaite-Morkune, Egle Zelbiene

**Affiliations:** Department of Emergency Medicine, Lithuanian University of Health Sciences, A. Mickevičiaus g. 9, Kaunas, LT-44307 Lithuania

## Abstract

**Background:**

Carbon monoxide (CO) poisoning is a serious yet frequently overlooked condition with diverse and nonspecific clinical presentations. The analysis of Lithuanian statistics reveals fluctuations in patient admissions and consultations through the poisoning center over a four-year period, with notable variations in fatality rates. Despite these trends, CO poisoning remains a significant public health concern due to its potential for severe long-term sequelae or death.

**Case:**

This case report focuses on two distinct presentations of CO poisoning in a young couple, illustrating the varied manifestations and outcomes of this toxic exposure. The first case describes a 23-year-old male presenting with altered consciousness and neurological symptoms, while the second case involves a 21-year-old pregnant female presenting with cardiovascular symptoms, including Takotsubo cardiomyopathy.

**Discussion:**

Highlights include the challenges in diagnosing CO poisoning; factors influencing the severity and symptoms of CO poisoning; potential complications; and considerations for hyperbaric oxygen therapy (HBO) in severe cases and pregnancy.

**Conclusion:**

These cases illustrate the importance of recognizing CO poisoning, advocating for oxygen therapy as the first-line treatment, and calling for further research to improve understanding, treatment, and prevention of this potentially fatal condition.

## Introduction

Carbon monoxide (CO) poisoning is a potentially fatal condition, often presenting with non-specific symptoms. It results from the inhalation of carbon monoxide, a colorless, odorless gas produced by the incomplete combustion of carbon-containing fuels, such as natural gas, gasoline, wood, and coal [1, 2]. CO binds to hemoglobin, causing tissue hypoxia and symptoms such as confusion, tachypnea, syncope, and chest pain. These symptoms can mimic conditions like influenza, food poisoning, myocardial ischemia, or stroke [1, 3] making diagnosis difficult especially if a history of exposure to the gas is unknown.

Analysis of Lithuanian statistics on patients with carbon monoxide poisoning from 2019 to 2022 reveals notable fluctuations in patient admissions, with the year 2019 reporting 41 cases (including 15 children), followed by a decrease in 2020 to 27 cases (including 4 children), then increase in 2021 to 36 cases (including 11 children), and a decline to 31 cases (including 7 children) in 2022. On average 34 patients are admitted to hospital yearly. Concurrently, consultations through the poisoning center exhibited variations, with 2019 recording 64 consults, decreasing to 31 in 2020, then rising to 73 in 2021, and stabilizing at 75 consults in 2022. Despite these trends, the fatality rates from carbon monoxide poisoning remained relatively low, with one reported death in both 2019 and 2020, a notable increase to four in 2021, and a return to zero fatalities in 2022 [4, 5].

This case report highlights the presentation, diagnosis, and treatment of two young patients exposed to carbon monoxide, emphasizing the diverse clinical manifestations and outcomes of this toxic exposure.

## Case 1: 23-Year-old male

**Fig. 1:**
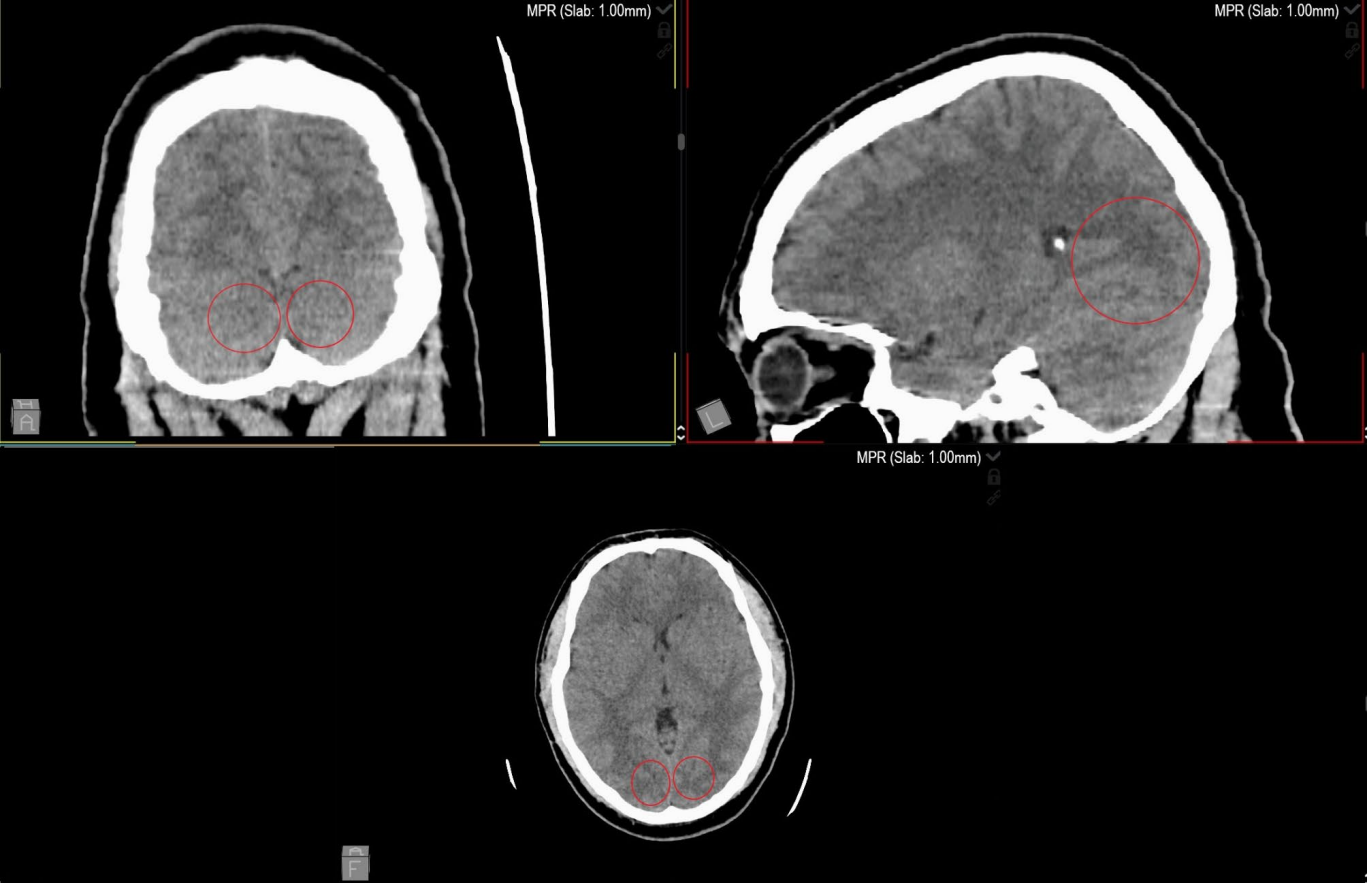
Male head CT with bilateral hypodensity in white matter, acute toxic leukoencephalopathy

A 23-year-old male presented to the emergency department (ED) with altered consciousness. The night before, he experienced symptoms of nausea, diarrhea, stomach pain, and general malaise. Emergency Medical Services (EMS) were called when he was found unconscious in his home. Upon EMS arrival, the patient had a Glasgow Coma Scale (GCS) score of 8/15, with stable vital signs and a strong smell of diesel fuel in the house. In the ED, his GCS improved to 10/15 (E-3, M-5, S-2), with stable vital signs. Venous blood gas analysis showed a pH of 7.43, pCO2 of 36.1 mmHg, pO2 of 22.4 mmHg, HCO3- of 23.8 mmol/l, base excess (BE) of -0.3, and elevated carboxyhemoglobin (COHb) at 28.8%. Head CT revealed bilateral deep white matter hypodensities, suggesting acute toxic leukoencephalopathy (see Fig. 1). The patient was diagnosed with acute CO poisoning, per our hospital protocol a toxicologist was consulted and recommended treatment included hyperbaric oxygen therapy or hyperventilation with 100% oxygen for at least 6 h, osmotic diuretics, and Vitamin B1 supplementation. Neurological examination revealed bilateral Babinski signs, consistent with central nervous system involvement. He was admitted to the intensive care unit (ICU) for further treatment.

In the ICU, following treatment with a non-rebreather mask, COHb levels initially decreased from 28.8 to 5.5% in 8 h. For brain protection, he was sedated but airway was left intact and received pain relief medications. The patient underwent two HBO sessions. The first session followed a protocol of 60 min at 2 ATA (atmospheres absolute), consisting of a 15-minute compression phase, a 30-minute plateau phase, and a 15-minute decompression phase. The second HBO session, conducted the next day, was interrupted during the initial minutes due to significant agitation. The COHb levels reduced to 2,8% after the first HBO session. His troponin levels were elevated at 1.47 ng/mL. Subsequent cardiac echocardiography revealed a left ventricular ejection fraction (EF) of approximately 50%, with reduced contractility observed in the apex of the heart. He was discharged after three days to a neurology unit.

After one month he received cardiology follow up - no abnormal findings, his cardiac echo showed no contractility issues, EF > 50proc.

At the two months follow up, patient reported memory issues, but neurological exam remained normal. Magnetic resonance imaging (MRI) was recommended but the patient refused further evaluation.

## Case 2: 21-Year-old pregnant female

The 21-year-old fiancée of the male patient, who was 14 + 5 weeks pregnant, also presented to the ED with mild chest pain, palpitations, and anxiety. Her arterial blood gas analysis showed a pH of 7.45, pCO2 of 13.8 mmHg, pO2 of 147 mmHg, HCO3- of 16.2 mmol/l, BE of -14.3, and COHb of 9.6%. The ECG showed sinus tachycardia, with a pulse of 100–125 beats/min. A rise in troponin levels (3.57 µg/l) was noted, and echocardiography revealed Takotsubo cardiomyopathy with reduced contractility in the apex of the heart. Additionally, fetal tachycardia up to 190 beats/min increased the pregnancy risk. The patient received oxygen therapy 15 L/min through a non-rebreather mask and was admitted to the cardiac ICU.

In the cardiac ICU, patient was managed with oxygen therapy through a mask, and her cardiac function improved. Her clinical condition improved, with cardiac function returning to normal, although concerns such as fetal tachycardia, mild abdominal pain persisted regarding fetal well-being. After initial concerns regarding fetal well-being, her condition stabilized, and she was discharged from the cardiac ICU.

After one month, the patient received gynecologic evaluation which identified a non-developing pregnancy, the US size of the fetus was around 14 weeks and it showed signs of maceration, although no anatomical abnormalities were observed. Pregnancy was terminated. She also received a cardiology follow-up which showed no abnormal findings.

At the two month follow up she also reported memory issues, but neurological exams remained normal. The female patient underwent a neuropsychological evaluation, revealing an elevated level of depression and anxiety, particularly concerning her health. Additionally, there was observed attentional instability, quick and impulsive actions leading to attention errors, and a somewhat inconsistent behavioral style. The MRI was recommended but the patient decided against it.

## Discussion

This case report on carbon monoxide (CO) poisoning in a young couple brings forth several important clinical considerations and insights into the diagnosis, management, and long-term implications of this potentially fatal condition.

### Clinical assessment and diagnosis

The diagnostic challenge in CO poisoning is underscored in these cases. The triad of symptoms consistent with CO poisoning, a history of exposure, and elevated carboxyhemoglobin (COHb) levels is crucial for diagnosis [6]. The diversity in clinical presentations observed in these cases exemplifies the hallmark variability of carbon monoxide (CO) poisoning. The male patient exhibited severe symptoms, including unconsciousness, which contrasted sharply with the female patient’s cardiovascular manifestations, notably Takotsubo cardiomyopathy (TTC). This variability highlights the critical need for a broad differential diagnosis and a heightened index of suspicion for CO poisoning, particularly in ambiguous cases, when patients present with non-specific, multisystem symptoms, or when multiple individuals from the same environment develop similar clinical features [7].Factors influencing severity and symptoms of CO poisoning include CO concentration and duration of exposure; pre-existing conditions that might impact oxygen carrying capacity (e.g., chronic lung disease); age; smoking; and pregnancy. CO binds more readily to fetal hemoglobin than to adult hemoglobin, potentially leading to fetal distress, low birth weight, or even miscarriage [8].Importance of Arterial Blood Gas and COHb Testing:

Given that no single symptom is pathognomonic for CO poisoning, arterial blood gas analysis and measurement of COHb levels are indispensable for diagnosis. The clinical diagnosis of acute CO poisoning should be confirmed by demonstrating an elevated carboxyhemoglobin level. COHb levels of more than 3–4% in nonsmokers and 10% in smokers are considered outside the expected physiological range [7].

Other tests which help determine need for admission and further follow up, also help determine spectrum of treatment lactate, creatine phosphokinase, troponin, and ECG [8, 9].

### Takotsubo cardiomyopathy

Myocardial injury in CO poisoning results from both tissue hypoxia and cellular-level damage [10]. Research indicates that younger patients, with fewer cardiac risk factors, are more likely to develop global hypokinesis of the left ventricle, which typically improves with treatment. In contrast, older patients, who have a higher prevalence of cardiac risk factors, often reveal underlying coronary artery disease due to CO poisoning’s disruption of the balance between oxygen supply and demand [10, 11]. Since such pathology, including Takotsubo cardiomyopathy (TTC), often resolves spontaneously, it may be prudent to monitor asymptomatic or mildly symptomatic patients with adequate organ perfusion, allowing the natural healing process to occur [12].

### CO poisoning in pregnancy

The management of CO poisoning in pregnancy, as seen in the female patient, presents unique challenges. The increased affinity of CO for fetal hemoglobin and slower clearance in the fetus necessitates prompt and effective treatment [13]. Eichorn described a case that showed a COHb measurement of 61% at fetal autopsy, although the mother had a measurement of 7% after just an hour of O2 treatment [14]. If HBO therapy is planned for the patient, the therapy duration should be longer for pregnant women than for non-pregnant women due to the slower dissociation of carboxyhemoglobin in the fetus [15]. Although this patient had a COHb level of 9.6%, the fetus was exhibiting tachycardia, suggesting fetal distress. However, there is no guarantee that HBO would have changed our patient’s fetal outcome. This case raises awareness about the risks to both the mother and the fetus, and the need for careful monitoring and management, including considerations for hyperbaric oxygen therapy (HBO).

### Hyperbaric oxygen therapy

The role of HBO in the treatment of CO poisoning is emphasized in the male patient’s case. HBO therapy is recommended for severe cases and specific scenarios like pregnancy, prolonged exposure, and high COHb levels (> 25%) [16]. While effective in reducing COHb levels it is believed to reduce mortality and neurological sequelae [17–19]. However, HBO therapy comes with potential complications such as lung damage, ear barotrauma, vision changes, hydrothorax, and may also cause seizures [20].

### Disposition

Recommendations for disposition are presented in Table 1.

**Table 1:** Recommendations for disposition [8, 9]

Symptom	Disposition	Comment
Minimal or no symptom	Home	Assess safety (is it safe to go back to environment, was it suicide attempt)
Headache, vomiting, elevated CO level	Home after symptom resolution	Administer 100% oxygen in ED, observe 4 h Assess safety issues
Ataxia, seizure, syncope, chest pain, focal neurologic deficit, dyspnea, ECG changes, metabolic acidosis	Hospitalize Consult for HBO therapy	Administer 100% oxygen in ED CO level, comorbid conditions (pregnancy, age, stability) should be considered before transport

### Long-term effects and delayed neuropsychological sequelae

CO poisoning can manifest long-term effects despite receiving treatment. Both patients exhibited long-term cognitive and psychological effects post-CO poisoning, highlighting the potential for delayed neuropsychological sequelae (DNS).

DNS can occur after CO poisoning, typically developing within weeks after an initial complete clinical recovery from acute poisoning. The reported incidence varies widely, ranging from 3 to 40%, due to the absence of established diagnostic criteria [21]. The most frequently described sequelae encompass a broad spectrum of neurological deficits, cognitive impairments, and affective disorders. DNS typically resolve gradually over the first few months but can be permanent in about 25% of cases [21].

HBO therapy has been utilized to prevent DNS, although there is limited evidence proving its effectiveness in improving patient outcomes in this area. Buckley et al. examined seven randomized trials to assess the efficacy of HBO compared to normobaric oxygen for preventing neurologic sequelae in patients with acute carbon monoxide poisoning [22]. Existing randomized trials did not establish whether the administration of HBO to patients with carbon monoxide poisoning reduces the incidence of adverse neurologic outcomes [22].

### Preventive measures

The report also emphasizes the importance of preventive measures against CO poisoning. According to available data, accidental CO poisoning in the US conservatively costs society over $1.3 billion, encompassing direct hospital expenses and lost earnings [23]. While data for Lithuania is limited, the consistent number of patients with this poisoning suggests that the issue persists.

One effective solution is the installation of CO sensors in houses. Although no extensive studies have been published to examine the effectiveness of CO alarms in preventing CO exposure, a comparable example is smoke alarms: homes with working smoke alarms have an 88% lower risk of fire-related injury or death [23]. A British cost-benefit analysis estimated CO alarms to be 75% effective [23]. Public education, especially for vulnerable groups like pregnant women, also plays a crucial role in prevention [24].

### Limitations and future research

Our case report has its limitations. The limitations in this case, including the patients’ refusal for further evaluation and the lack of definitive answers regarding the optimal HBO therapy for pregnant patients, highlight the need for more research. Furthermore, there is a lack of large-scale studies on the prevention of CO poisoning. These areas highlight the need for more comprehensive research on the long-term cognitive effects of CO poisoning.

## Conclusion

This case report emphasizes the importance of recognizing CO poisoning, which can present diverse clinical manifestations.Oxygen therapy remains the first-line treatment for CO poisoning, and hyperbaric oxygen therapy should be considered in indicated situations, especially when the patient is pregnant. Additionally, this case report underscores the necessity for long-term follow-up, even when patients appear to have recovered.

## Data Availability

No datasets were generated or analysed during the current study.
